# Urbanization may affect the incidence of urolithiasis in South Korea

**DOI:** 10.1186/s40064-016-3554-x

**Published:** 2016-10-28

**Authors:** Se Young Choi, Seo Yeon Lee, Byung Hoon Chi, Jin Wook Kim, Tae-Hyoung Kim, In Ho Chang

**Affiliations:** 1 Department of Urology, Chung-Ang University Hospital, Seoul, South Korea; 2 Department of Urology, Myongji Hospital, Seonam University College of Medicine, Goyang, South Korea

## Abstract

**Background:**

We evaluated the different climatic factors in urban and rural areas that may affect the incidence of urolithiasis. Nationwide data on urolithiasis were acquired from Health Insurance Review and Assessment Service between 2009 and 2013. Information on age, gender, date of diagnosis, geographic region and daily weather data from all weather stations was collected. The data were grouped by population density and substituted into the lag period model. The primary outcome was the incidence rate in each region. The secondary outcomes were differences between groups and relative risks (RRs) of climatic factors. The tertiary outcome was RRs of urolithiasis presentation cumulated over a 20-day lag period associated with the mean daily temperature.

**Results:**

The incidence rates of urolithiasis tended to increase annually in most regions from 2009 to 2013. The urban group showed a higher mean temperature, lower amount of rainfall, higher wind speed and lower mean relative humidity than the rural group (p < 0.001). The urban group showed significant RRs of temperature (1.013, 95% confidence interval [CI] 1.009–1.017, p < 0.001), wind speed (0.979, CI 0.973–0.986, p < 0.001), humidity (0.995, CI 0.994–0.996, p < 0.001), and sunshine (0.992, CI 0.988–0.996, p < 0.001). The rural group showed significant RRs of wind speed (0.980, CI 0.968–0.992, p = 0.002) and humidity (0.998, CI 0.996–0.999, p = 0.007). In the urban area, RRs increased gradually with increasing temperature.

**Conclusions:**

Regional differences in climatic factors, especially temperature, may provoke a gap in urolithiasis events between the urban and rural areas.

**Electronic supplementary material:**

The online version of this article (doi:10.1186/s40064-016-3554-x) contains supplementary material, which is available to authorized users.

## Background

The incidence of symptomatic events such as renal colic reaches its peak in summer which is characterized by high temperature and sunlight exposure (Prince and Scardino 1960). The hypothesis that dehydrated, concentrated, and acidified urine promote supersaturation and nucleation may be the first steps of stone formation (Prince and Scardino 1960; Eisner et al. 2012). Geographical location influences the formation of urinary stones. Various regions of the world displayed different prevalence such as 1–5% in Asia, 5–9% in Europe, and 13% in North America (Ramello et al. 2000). In addition, a hypothesis that urban heat islands (UHIs) with elevated temperatures in the urban area compared to the rural area can cause an increase in the incidence of urolithiasis was proposed, but a few studies support this hypothesis (Goldfarb and Hirsch 2015).

Recently, the concept of lag periods between temperature exposure and occurrence was introduced, and it did not assess the temperature during 1 day of the incidence but it assessed cumulative exposure to temperature during serial periods until the outbreak (Tasian et al. 2014). This model may help to fill the gap between stone formation and visit to the hospital due to symptoms.

In Republic of Korea (ROK) only one National Health Insurance system covers over 50 million people, which almost corresponds to the national population (Moon 2014). The aim of study was to evaluate the effect of meteorological factors that may affect the age and sex adjusted urolithiasis incidence and to assess whether UHIs can cause an increase in the incidence of urolithiasis using the nationwide data with the lag period model.

## Methods

### Data sources

Nationwide data on urolithiasis from January 2009 to December 2013 were acquired from Health Insurance Review and Assessment Service (HIRA). The codes N20 (calculus of kidney and ureter), N21 (calculus of lower urinary tract), N22 (calculus of urinary tract in diseases classified elsewhere), and N23 (unspecified renal colic) were included. Patients under age 18 were excluded because pediatric urolithiasis was mostly correlated with metabolic abnormalities (Penido and Tavares Mde 2015). Revisits within 30 days after the first visit were also excluded because it was considered as the same event.

Daily weather data were acquired from National Climate Data Service System (NCDSS, http://sts.kma.go.kr/), and population data were obtained from Korean Statistical Information Service (KOSIS, http://kosis.kr/).

### Geography, climate and administrative divisions of ROK

ROK lies between latitudes 33° and 39° N, and longitudes 124° and 130° E. ROK has a humid continental and subtropical climate. Summer is affected by the East Asian monsoon and has a short rainy season which starts in late June and ends in the end of July. ROK has four distinct seasons; spring, summer, autumn and winter. The major administrative divisions of ROK include nine provinces (Gyeonggi, Gangwon, Chungbuk, Chungnam, Jeonbuk, Jeonnam, Gyeongbuk, Gyeongnam, and Jeju) and seven metropolitan cities (Seoul, Busan, Incheon, Daegu, Gwangju, Daejeon, and Ulsan).

### Incidence rate (IR) definition

In this study, the IRs were calculated using the formula of the number of urolithiasis cases over the population in 100,000 person-years for each year. The cases were stratified by sex and age categories (every 10 years of age, data not shown) and the IRs were adjusted for age and sex using a direct standardized method in each region.

### Statistical methods

Cities and provinces were divided into group A (≥1000 people/km^2^) and group B (<1000 people/km^2^), respectively based on the population density to evaluate the difference between the urban and rural areas. Continuous variables of climatic factors were checked by the Kolmogorov–Smirnov test to confirm the normality of the distribution (data not shown). Nonparametric variables were compared using the Mann–Whitney test.

Distributed lag nonlinear models (DLNMs) were employed to document climatic factors associated with urolithiasis presentation, given as relative risks (RRs) with 95% confidence intervals (95% CI) (Tasian et al. 2014). The RRs of urolithiasis event were evaluated over the distribution of MTs compared to a MT of 13 °C, which is the MT of ROK. We summed the estimated risks for each lag day to evaluate the cumulative RR for the event related to MTs during the 20-day period after temperature exposure (Boscolo-Berto et al. 2008).

Values of p < 0.05 were considered statistically significant. Analyses and figure measurements were performed with R (version 3.1.3; R Project for Statistical Computing; http://www.r-project.org/).

## Results

The total number of urolithiasis presentations from January 2009 to December 2013 was 1,452,671 (Additional file 1). The number of cases and IR increased every year from 2009 to 2013. The mean age [±standard deviation (SD)] was 47.7 ± 13.9 years for males, 50.8 ± 14.2 years for females, and 48.8 ± 14.1 years for both genders, and the ratio of urolithiasis incidence in males and females was 6.5:3.5.

The regions were divided by population density (Additional file 2). Group A included all the cities and the capital area Gyeonggi province. The range of population density was from 1045 (Ulsan) to 16,558 (Seoul). Group B contained the remaining provinces. The range of population density was from 89 (Gangwon) to 306 (Gyeongnam).

**Fig. 1:**
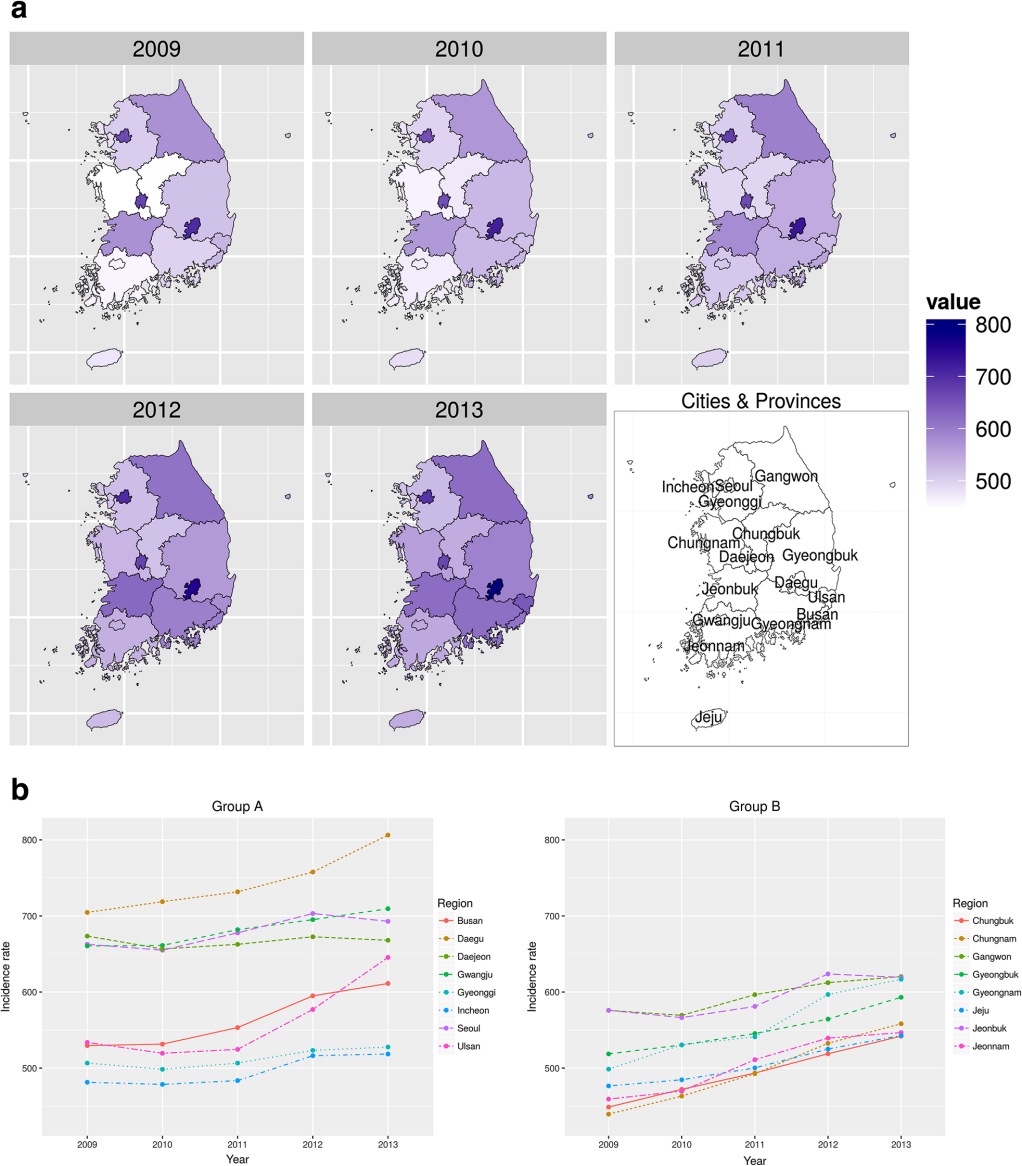
**a** Maps of South Korea with age-sex adjusted incidence rates of urinary stone. **b** Trends of age-sex adjusted incidence rates in both groups. Age-sex adjusted incidence rates of urinary stone per 100,000 population tended to increase annually in most regions from 2009 to 2013

Age-sex adjusted IRs for urolithiasis per 100,000 population tended to increase annually in most regions from 2009 to 2013. The trends are shown in the form of maps (Fig. 1a) and graphs (Fig. 1b).

Group A showed higher daily mean temperature (MT), lower amount of rainfall, higher wind speed, and lower mean relative humidity (RH) than group B (Table 1). There were no differences in the mean sea level pressure and the amount of sunshine between groups A and B. The correlations among climatic factors were significant except for the correlation between rainfall and wind speed. The linear formula between time and MT was as follows (p < 0.001): Temperature (°C) = 0.000755 × time (day) + 11.32.

**Table 1:** Daily measured climatic factors acquired from the weather station of each region

	Total	A	B	p value
Mean temperature (°C, range)	13.12 ± 9.26 (−14.56 to 33.10)	13.32 ± 10.18 (−14.56 to 33.10)	12.91 ± 8.23 (−14.50 to 30.66)	*<0.001*
Rainfall (mm)	4.60 ± 14.38	3.99 ± 15.00	5.21 ± 13.69	*<0.001*
Wind speed (m/s)	2.53 ± 1.80	2.76 ± 2.26	2.29 ± 1.14	*<0.001*
Mean sea level pressure (hPa)	1016.00 ± 9.33	1015.97 ± 11.47	1016.03 ± 6.53	0.647
Mean relative humidity (%)	66.18 ± 14.45	64.04 ± 16.35	68.32 ± 11.88	*<0.001*
Sunshine (h)	5.78 ± 3.54	5.76 ± 3.82	5.79 ± 3.24	0.765

Italic values indicate significance of p value (p < 0.05)

The RRs of urolithiasis IR are displayed in Table 2. Group A showed significant results for MT, wind speed, RH, and sunshine (1.013 [95% CI 1.009–1.017], 0.979 [95% CI 0.973–0.986], 0.995 [95% CI 0.994–0.996], and 0.992 [95% CI 0.988–0.996], respectively). Group B showed significant results for wind speed and RH (0.980 [95% CI 0.968–0.992] and 0.998 [95% CI 0.996–0.999], respectively).

**Table 2:** Relative risks of climatic factors for urolithiasis incidence rate

	A		B	
	RR (95% CI)	p value	RR (95% CI)	p value
Mean temperature	1.013 (1.009–1.017)	*<0.001*	1.000 (0.997–1.003)	0.965
Rainfall	1.000 (0.999–1.001)	0.770	1.000 (0.999–1.001)	0.949
Wind speed	0.979 (0.973–0.986)	*<0.001*	0.980 (0.968–0.992)	*0.002*
Mean sea level pressure	1.000 (0.999–1.001)	0.435	0.997 (0.994–1.000)	0.088
Mean relative humidity	0.995 (0.994–0.996)	*<0.001*	0.998 (0.996–0.999)	*0.007*
Sunshine	0.992 (0.988–0.996)	*<0.001*	0.996 (0.990–1.002)	0.171

Italic values indicate significance of p value (p < 0.05)

**Fig. 2:**
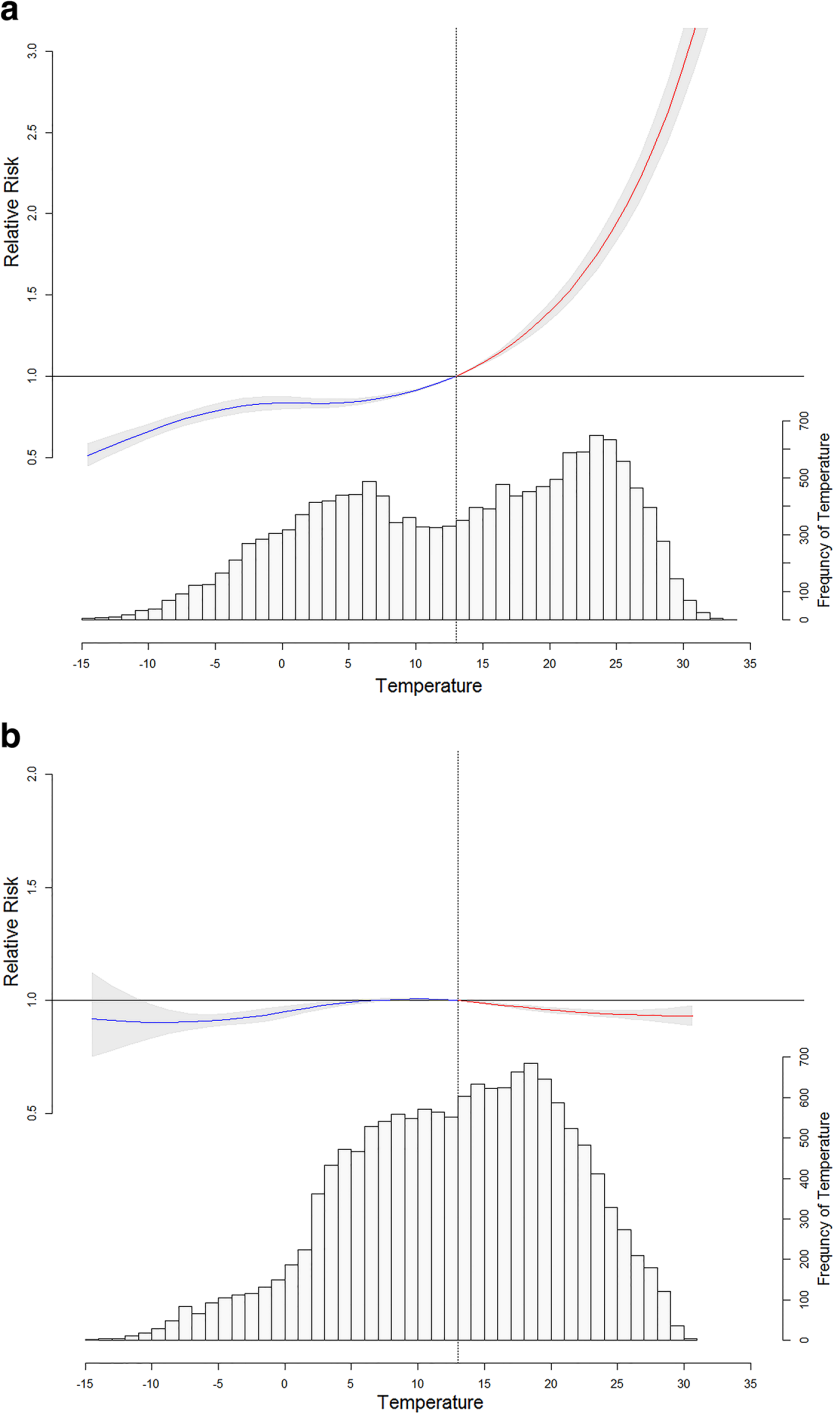
Relative risks of urolithiasis presentation cumulated over a 20-day lag period associated with the mean daily temperature relative to 13 °C in group A (**a**) and group B (**b**) from 2009 to 2013 and the surrounding *gray areas* are the 95% confidence interval

Groups A and B showed different aspects in RRs of urolithiasis event cumulated over a 20-day lag period associated with MT compared to 13 °C (Fig. 2a, b). The RR in group A almost resembled a J-shaped curve, but the RR in group B was almost consistent regardless of the change in temperature. The frequency of MT in group B was more centralized than the frequency of MT in group A. In group A, RRs were 2.088 (95% CI 1.960–2.224) at 26 °C and 0.836 (95% CI 0.795–0.879) at 0 °C. In group B, RRs were 0.937 (95% CI 0.917–0.958) at 26 °C and 0.950 (95% CI 0.923–0.977) at 0 °C.

## Discussion

In our study, various urolithiasis IRs and different climatic factors were presented depending on the regions, but the most important factor was MT in the high population density group compared to the low population density group. Among the significant RRs in the urban group, wind speed and RH also affected the rural group and the amount of sunshine did not show a significant difference between the urban and rural groups. Therefore, MT may be responsible for the difference in urolithiasis incidence between the urban and rural groups.

In the urban group, RRs increased gradually with increasing temperature and the RR almost doubled with a twofold increase in the temperature compared to that at 13 °C. Urolithiasis prevalence in the southeastern United States (USA) is almost 50% higher than that in the northeastern USA which has a 8 °C lower MT and an increase in 1 °C will cause a 4.2% increase in the urolithiasis risk (Brikowski et al. 2008). Another study that used the multivariate autoregressive integrated moving average (ARIMA) model showed that temperature has a strong correlation with calculi presentation rate in New York City (Sirohi et al. 2014). Similarly, among various climatic factors, the association between temperature and urinary stone event is well known.

The importance of temperature in resultant urolithiasis implies two things. First, urbanization may affect the prevalence of urolithiasis. The urban population exceeded the rural population all over the world from 2008 and urbanization is a worldwide phenomenon (Desa 2010). A decrease in the green area in city causes elevation of the temperature due to UHIs compared to the rural area, but water in the rural surface absorbs the heat that converts it to cause evaporation (Goldfarb and Hirsch 2015). Elevated temperatures influence various diseases like cardiovascular disease, asthma etc (O’Neill and Ebi 2009). In addition, many studies have demonstrated the association between hotter season or months and urolithiasis events (Boscolo-Berto et al. 2008; Brikowski et al. 2008; Sirohi et al. 2014). In our study, the urban group included cities and the capital area that have a high population density; hence, most of them are composed of urban areas. The temperature in the urban group was higher than that in the rural group, and the average IR in the urban group was higher than the average IR in the rural group. Meanwhile, there are a few studies comparing urolithiasis presentation according to regional differences. In the USA, there was no difference in the pediatric kidney stone incidence between the urban and rural areas (Sas et al. 2010). The authors did not explain the reason for this occurrence. All their subjects were children; hence, their results cannot be directly compared to our results.

Second, global warming can also increase urolithiasis events. The greenhouse gas concentrations increased surface temperatures from 1.5 to 4.5 °C during the twenty-first century (Meinshausen et al. 2011). The effects of global warming are observed even in ROK such as an increase in atmospheric carbon dioxide and temperatures (Oh et al. 2001). In the USA, the prevalence of urolithiasis increased from 3.6 to 5.2% when the annual MT increased by 0.5 °C from 1976 to 1994 (Stamatelou et al. 2003). Brikowshi et al. (2008) predicted that 56% of the USA population would live in high-risk stone areas and there would be additional 1.6–2.2 million cases based on the global warming model until 2050. According to our linear formula between time and temperature, there was an increase of 1.38 °C during 5 years and there were additional 30,918 cases in 2013 compared to those in 2009. This increasing trend was also observed in another Korean nationwide study and the annual increase rate was calculated as 0.3% using the ARIMA model from 2006 to 2010 (Park et al. 2015).

The main finding of our study is that the pattern of occurrence of urolithiasis is affected by the regional difference. In our study, RH and wind speed affected urolithiasis incidence in the urban and rural areas and sunshine affected urolithiasis incidence only in the urban area. In other studies, the associations between humidity and urolithiasis are debatable (Tasian et al. 2014; Sirohi et al. 2014). Humidity does not have an effect on people’s health as much as temperature, but other factors could conceal the effect of humidity (Barnett et al. 2010). In addition, humidity may cause more severe problems in special zones of the city in which people who are vulnerable to heat like senior citizens live (Kenney and Hodgson 1987). Low RH environment induces an increase in transepidermal water loss (Kenney and Hodgson 1987). This can be related to the hypothesis of dehydration and stone formation. Regions with vegetation have higher humidity and the influence of humidity on temperature can differ depending on the urban conditions (Hass et al. 2016). Increased wind speed may cool the people down and influence solar radiation and temperature on humans (Thorsson et al. 2007). Sunlight exposure is correlated with increased urolithiasis in many studies (Prince and Scardino 1960). However, opposite results in our study might be due to the fact that the rainy season is usually in summer in ROK and sunshine has decreased in cities during the past 46 years because of anthropogenic activities and air pollution (Fu et al. 2015).

Interestingly, in our study, urolithiasis incidence increased during the 2009–2013 period. This increasing trend could be explained by the increase in hospital utility in Korea. The mean number of hospital utility episodes per person increased from 16.04 in 2006 to 18.59 in 2010 (Moon 2014). Another explanation is that nonenhanced computed tomography (CT) has replaced intravenous urography (IVU) as an imaging tool for confirming the diagnosis of urolithiasis (Lee et al. 2015). CT has a higher detection rate of urolithiasis including radiolucent stones and small stones compared with IVU (Lee et al. 2015). CT has been described as the best imaging tool for confirming the diagnosis of urolithiasis, and CT may have affected the annual increase rate of urolithiasis in our study.

There are some limitations to this study. First, lag periods do not directly indicate the duration of stone formation. Lag periods indicate the association between daily temperatures and urolithiasis events. We observed an increasing RR of urolithiasis after lag periods of high temperature. High temperature might cause early occurrence of the urolithiasis event. High temperature could cause the previously formed asymptomatic stones to become symptomatic or aggravate new stone nucleation. Second, there may be confounding factors due to the difference in urban and rural areas. The difference may be applicable to age, lifestyle, diet, income, activity and occupation, which may be extrinsic factors of urolithiasis (Ramello et al. 2000; Scales et al. 2012). Unfortunately, no information was provided about dietary habits of the population of different areas in our study and it is very difficult to estimate the lifestyle and diet habits for whole population in South Korea. Diet is the other main determinant of stone formation and it is reasonable to suppose that people living in the rural area could have different dietary habits than people living in the towns who often did not take their meals at home. Also age distribution could be different in different areas and it could be that populations in the urban areas were younger and more prone to stone formation. Air-conditioned indoor condition and heat exposure outdoor condition in the same region can have other effects on urolithiasis (Ramello et al. 2000; O’Neill and Ebi 2009). Lastly, the inclusion and the exclusion criteria could be a source of bias, which are patients with chronic urolithiasis which come at the control after more than 30 days and there are persons with congenital urolithiasis which is not dependent on the climatic area. Unfortunately, no information was provided from Health Insurance Review and Assessment Service (HIRA) in our study. More studies in different populations are needed to confirm the physiology of urolithiasis caused by change in climatic factors under the control of other extrinsic factors.

## Conclusions

We conclude that regional differences in climatic factors, especially temperature, may provoke a gap in urolithiasis events. The regions were divided based on the criteria of population density. In the urban area, RRs increased gradually with increasing temperature. This study can help predict urolithiasis events under the condition of urbanization or global warming.

## Additional files

**Additional file 1.** Urinary stone incidence rate per 100,000 population and basic characteristics.

**Additional file 2.** Regions grouped by population density.
