# Supplementary material for: Urbanization may affect the incidence of urolithiasis in South Korea
Source: Springerplus. 2016 Oct 28;5(1):1891. doi: 10.1186/s40064-016-3554-x (PMC5084141; doi:10.1186/s40064-016-3554-x)
Supplement: Supplementary file 1 — Additional file 1. Urinary stone incidence rate per 100,000 population and basic characteristics. [file 40064_2016_3554_MOESM1_ESM.pdf]

Supplementary material 1. Urinary stone incidence rate per 100,000 population and basic characteristics

		2009 (n=276897)	2010 (n=278301)	2011 (n=287719)	2012 (n=301939)	2013 (n=307815)
Total		556.32	556.75	572.73	598.44	607.56
Region	Seoul (City)	660.79 (24.4%)	652.90 (23.8%)	675.43 (23.7%)	700.51 (23.3%)	689.90 (22.4%)
	Busan (City)	534.82 (6.8%)	536.93 (6.8%)	558.17 (6.8%)	599.59 (6.9%)	615.18 (7.0%)
	Incheon (City)	482.74 (4.7%)	479.47 (4.7%)	484.67 (4.7%)	517.55 (4.8%)	519.40 (4.8%)
	Daegu (City)	706.61 (6.4%)	721.04 (6.4%)	734.17 (6.4%)	760.34 (6.3%)	808.89 (6.5%)
	Gwangju (City)	648.35 (3.4%)	649.73 (3.4%)	670.12 (3.4%)	683.03 (3.3%)	697.45 (3.3%)
	Daejeon (City)	669.19 (3.6%)	652.83 (3.5%)	659.47 (3.5%)	667.73 (3.3%)	663.53 (3.3%)
	Ulsan (City)	538.72 (2.2%)	525.37 (2.1%)	529.08 (2.1%)	581.01 (2.2%)	650.15 (2.4%)
	Gyeonggi (Province)	506.57 (21.0%)	498.17 (20.9%)	506.70 (20.8%)	523.89 (20.8%)	528.16 (20.8%)
	Gangwon (Province)	577.58 (3.2%)	571.12 (3.1%)	600.10 (3.1%)	615.58 (3.1%)	623.88 (3.1%)
	Chungbuk (Province)	447.86 (2.5%)	471.24 (2.6%)	492.90 (2.6%)	518.19 (2.7%)	541.86 (2.8%)
	Chungnam (Province)	436.45 (3.2%)	459.73 (3.4%)	489.80 (3.4%)	529.28 (3.5%)	555.56 (3.7%)
	Jeonbuk (Province)	574.01 (3.8%)	564.51 (3.8%)	578.34 (3.8%)	621.92 (3.8%)	618.36 (3.7%)
	Jeonnam (Province)	457.55 (3.2%)	469.72 (3.2%)	510.85 (3.2%)	538.61 (3.4%)	546.48 (3.4%)
	Gyeongbuk (Province)	515.49 (5.0%)	528.21 (5.1%)	543.72 (5.1%)	561.80 (5.0%)	590.79 (5.1%)
	Gyeongnam (Province)	499.11 (5.9%)	531.31 (6.2%)	541.80 (6.2%)	597.89 (6.5%)	618.52 (6.7%)
	Jeju (Province)	474.71 (1.0%)	483.22 (1.0%)	499.76 (1.0%)	524.94 (1.0%)	542.91 (1.0%)
Age		47.7±14.0	48.3±14.0	48.9±14.0	49.3±14.1	49.7±14.2
Sex	Male	722.94 (65.1%)	721.69 (64.9%)	748.26 (65.4%)	783.58 (65.5%)	804.91 (66.2%)
	Female	389.12 (34.9%)	391.41 (35.1%)	396.91 (34.6%)	413.17 (34.5%)	410.25 (33.8%)
