# Supplementary material for: Urbanization may affect the incidence of urolithiasis in South Korea
Source: Springerplus. 2016 Oct 28;5(1):1891. doi: 10.1186/s40064-016-3554-x (PMC5084141; doi:10.1186/s40064-016-3554-x)
Supplement: Supplementary file 2 — Additional file 2. Regions grouped by population density. [file 40064_2016_3554_MOESM2_ESM.pdf]

Supplementary material 2. Regions grouped by population density

Group	A	B
Population density (people per km^2)	≥1000	<1000
Region	Seoul<br>Busan<br>Incheon<br>Daegu<br>Gwangju<br>Daejeon<br>Ulsan<br>Gyeonggi	Gangwon<br>Chungbuk<br>Chungnam<br>Jeonbuk<br>Jeonnam<br>Gyeongbuk<br>Gyeongnam<br>Jeju
Total Incidences (cases)	1036062	416609
Average incidence rate (per 100,000 population)	612.90	537.37
